# Supplementary material for: Engineering sulfonate group donor regeneration systems to boost biosynthesis of sulfated compounds
Source: Nat Commun. 2023 Nov 10;14:7297. doi: 10.1038/s41467-023-43195-1 (PMC10638397; doi:10.1038/s41467-023-43195-1)
Supplement: Supplementary file 1 — Supplementary Information [file 41467_2023_43195_MOESM1_ESM.pdf]

Engineering sulfonate group donor regeneration systems to boost biosynthesis of sulfated compounds

Xu *et al.*

Supplementary Table 1. Expression level of the enzymes applied in the PAPS regeneration system.

Enzyme	Cultivation condition	OD_{600}	Expression level (g/L)
ASAK	30℃ for 15 h	13	0.38 ± 0.03
*Kp*CysQ	30℃ for 15 h	13	0.62 ± 0.04
$PPK2^{s.e}$	30℃ for 15 h	14	0.51 ± 0.03
$ATPS^{S}$	30℃ for 15 h	13	0.32 ± 0.02

The strains were cultivated in TB medium at the designated temperature, purified using Ni-NTA columns, and quantified through a modified Bradford protein assay.

Supplementary Table 2. Kinetic parameters of Stf0$^{R143C/W154N}$ and AST IV$^{R132Q/V139G/S140I}$.

Enzyme	Substrate	K_m (mM)	K_{cat} (h^{-1})	k_{cat}/K_m ($h^{-1}\cdot mM^{-1}$)
Stf0$^{R143C/W154N}$	APS	1.04 ± 0.17	35.12 ± 0.94	33.77
	PAPS	0.78 ± 0.14	36.10 ± 0.98	46.28
AST IV$^{R132Q/V139G/S140I}$	APS	2.12 ± 0.39	38.76 ± 2.47	18.28
	PAPS	1.27 ± 0.13	51.93 ± 3.00	40.89

Kinetic parameters were determined for mutant AST IV$^{R132Q/V139G/S140I}$ and Stf0$^{R143C/W154N}$ using 0 – 10 mM APS or PAPS. The reaction was initiated by addition of enzyme at the following concentrations: 10 mM $MgCl_2$, 1.0 g/L AST IV$^{R132Q/V139G/S140I}$ and Stf0$^{R143C/W154N}$ at 35°C for 15 min. At selected time points, the reaction was quenched in boiling water. The supernatant was analyzed by HPLC/MS.

Supplementary Table 3. Comparison of different sulfonate group regeneration systems.

No.	Enzyme addition	Expression host	Substrates addition*	Byproducts accumulation	Capacity	Catalytic condition	Reference
1	2 enzymes	*E. coli*	PNPS (US$199/g)<br>PAP (US$1,5099/g)	PNP (toxic)	3.14 g/L<br>Triacetylchitotriose<br>95% yield	Room temperature<br>3 days	[1]
2	5 enzymes	*E. coli*	ATP ($15.4/g), $MgSO_4$,<br>PNPS	PNP (toxic),<br>ADP, Pi	0.1 g/L heparosan	37°C<br>40 h	[2]
3	7 enzymes	*E. coli* or commercial	ATP, $MgSO_4$, PEP (US 386/g)	Pyr, Pi,	2 g/L oligosaccharide<br>84% yield	25°C<br>2 days	[3]
4	Engineering *E. coli*	*E. coli*	Culture medium	/	5 µg chondroitin<br>37% yield	/	[4]
5	Whole cell catalysis	*E. coli*	Culture medium, $polyP_6$	/	2 g/L *p*-coumaric acid<br>86% yield	/	This study

*Price was obtained from https://www.sigmaaldrich.com

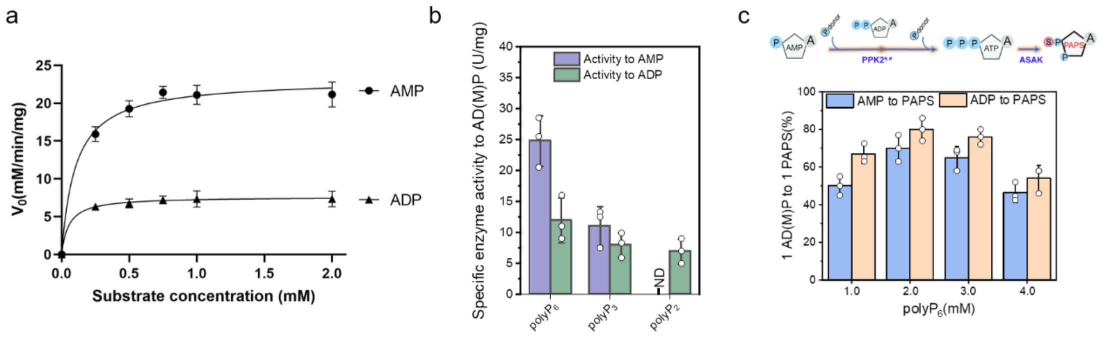

Supplementary Fig. 1. Enzymatic properties of polyphosphokinase PPK2$^{s.e}$.

a: Kinetic analysis of the AMP phosphorylase and ADP phosphorylase of PPK2$^{s.e}$.
b: Activity analysis of PPK2$^{s.e}$ by using polyphosphates with different degrees of polymerization. ND means not detected.
c: Cascade reaction of PAPS synthesis from AMP or ADP.
All the data are expressed as the mean ± S.D. from three (n = 3) biologically independent replicates. Source data are provided as a Source Data file.

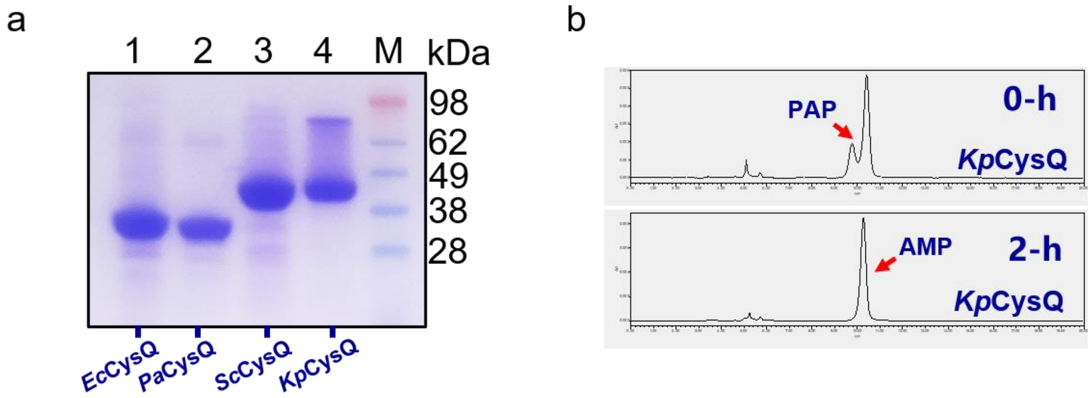

Supplementary Fig. 2. The purification and enzymatic property analysis of CysQ.

a: SDS-PAGE of purified CysQ from different species. Three independent experiments were performed, with similar results. Source data are provided as a Source Data file.
b: HPLC analysis of the PAP dephosphorylation catalyzed by *Kp*CysQ.

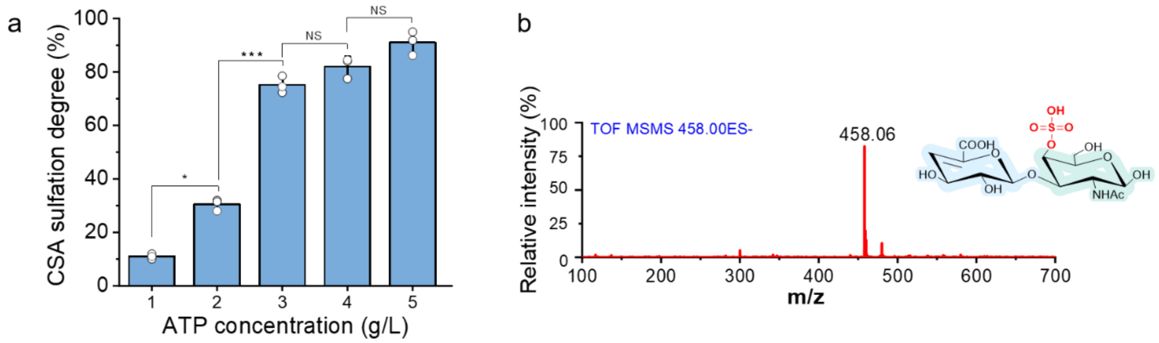

Supplementary Fig. 3. Optimization of ATP concentration to sulfonate chondroitin and mass spectrometry analysis.

a: Optimization of ATP concentration for sulfonating chondroitin couple with the PAPS regeneration system. Significance (*P* value) was evaluated by two-sided *t*-test, *, **, *** denote *P* value < 0.05, <0.01, <0.001, respectively, NS, not significant (P > 0.05).
b: Mass spectrum of chondroitin disaccharide A disaccharide.
All the data are expressed as the mean ± S.D. from three (*n* = 3) biologically independent replicates. Source data are provided as a Source Data file.

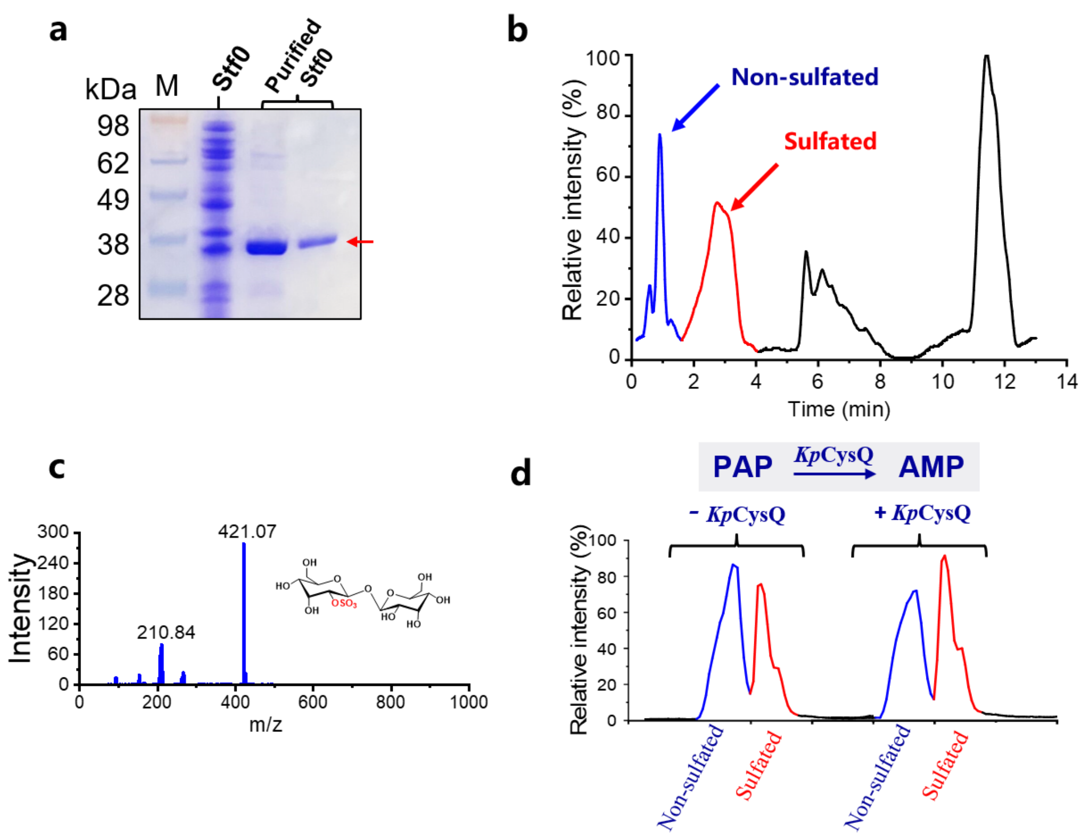

Supplementary Fig. 4. Sulfonation of trehalose, detection, and optimization.

a: SDS-PAGE of the Stf0 and purified Stf0. Three independent experiments were performed, with similar results.
b: Separation and identification of trehalose and trehalose-2-sulfate using total ion chromatography by HPLC/MS.
c: Mass spectrum of trehalose-2-sulfate.
d: Enhancing the Stf0 enzyme activity by introducing *Kp*CysQ.
Source data are provided as a Source Data file.

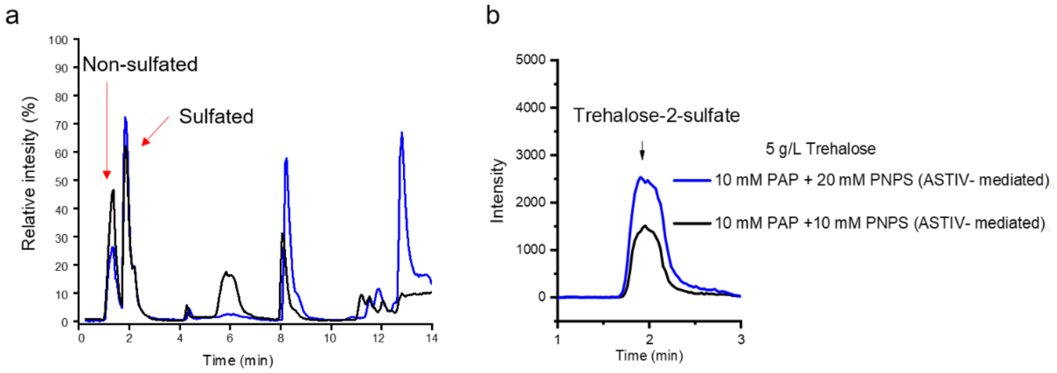

Supplementary Fig. 5. Sulfonation of trehalose based on the AST IV-catalyzed co-factor recycling system.

Separation and identification of trehalose and trehalose-2-sulfate via total ion chromatography (a) and quantification of trehalose-2-sulfate (b) using HPLC/MS based on the AST IV-catalyzed co-factor recycling system[6]. Trehalose was sulfated by the AST IV enzyme with varying concentrations of PNPS as the sulfate donor. Source data are provided as a Source Data file.

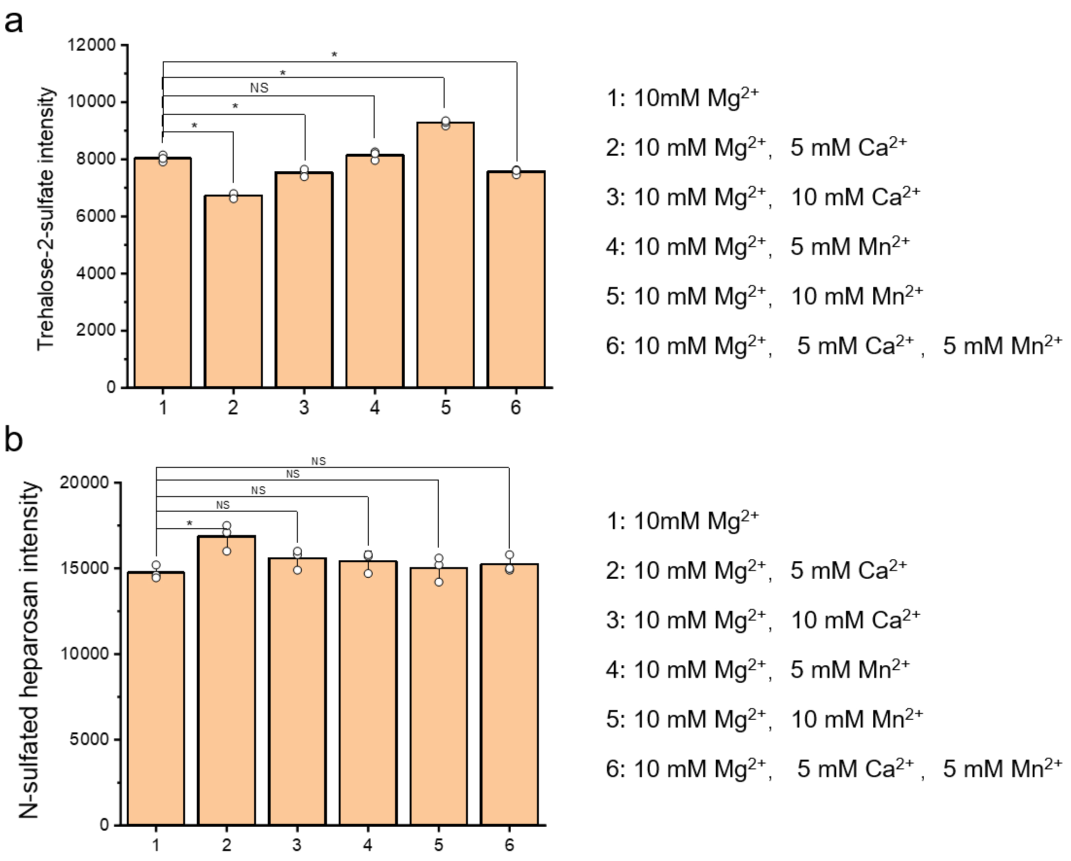

Supplementary Fig. 6. Investigating the impact of adding Ca^{2+} and Mn^{2+} on PAPS regeneration efficiency.

Determination of sulfation trehalose (a) and *N*-heparosan (b) using the PAPS regeneration system with the addition of Ca^{2+} and Mn^{2+}.

Significance (*P* value) was evaluated by two-sided *t*-test, *, **, *** denote *P* value < 0.05, <0.01, <0.001, respectively, NS, not significant (*P* > 0.05).

All the data are expressed as the mean ± S.D. from three (*n* = 3) biologically independent replicates. Source data are provided as a Source Data file.

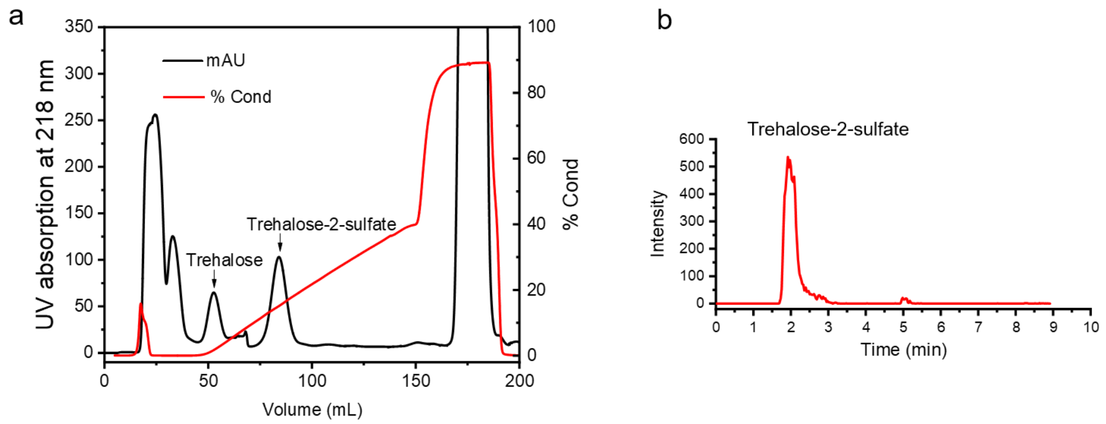

Supplementary Fig. 7. Purification and purity identification of trehalose-2-sulfate.

(a) Anion-exchange chromatograms of depolymerization products linearly eluted with 0–1000 mmol/L NaCl on a Q HP column. (b) LC-MS spectra of trehalose-2-sulfate. The trehalose-2-sulfate was purified with anion exchange chromatography (HiTrap 16/10 Q FF column). The product was eluted on ÄKTA pure chromatography system with buffer A (20 mM Tris-HCl, pH 8), and buffer B (20 mM Tris-HCl containing 1000 mM NaCl) at a flow rate of 3 mL/min.

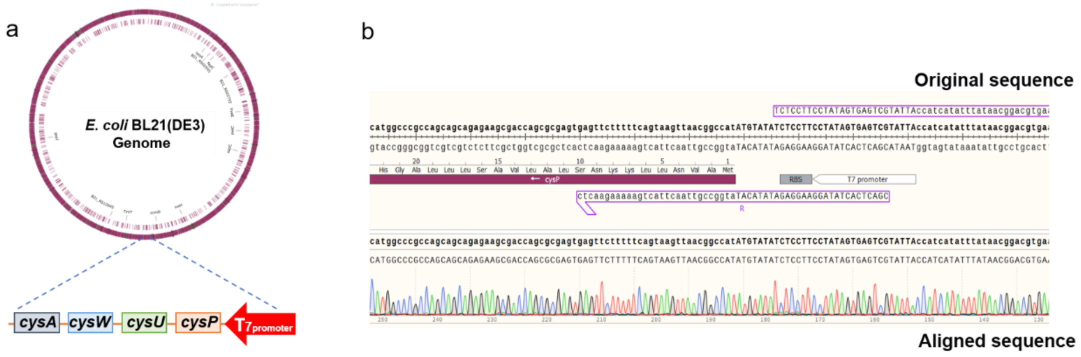

Supplementary Fig. 8. Genome-enhanced expression of *cysPUWA* by T7 promoter.

a: Schematic diagram of enhanced genome expression of *cysPUWA* by T7 promoter.

b: Sequence alignment validation of genome-enhanced expression of *cysPUWA* by T7 promoter.

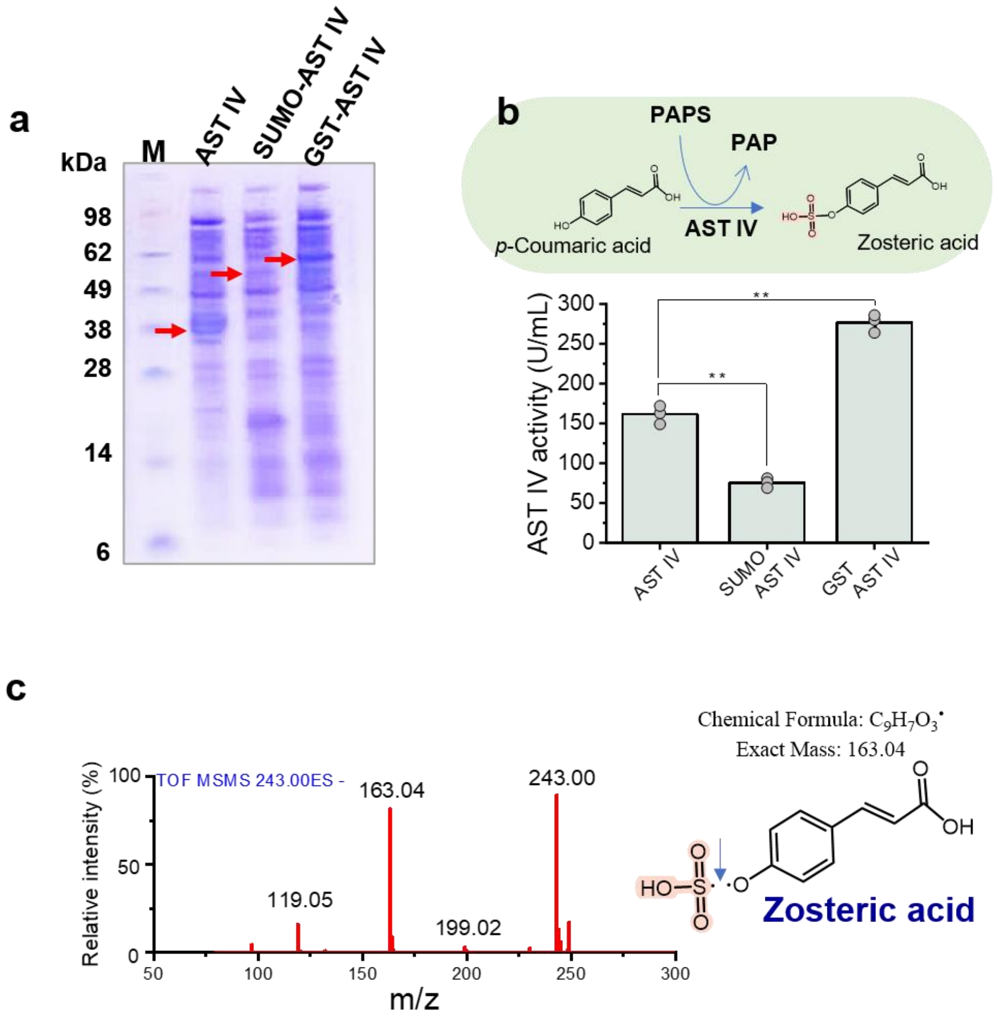

Supplementary Fig. 9. Synthesis of zosteric acid using AST IV.

a: SDS-PAGE of the AST IV with different N-terminal fusion with tags. Three independent experiments were performed, with similar results.
b: Enzyme activity assay of AST IV with different N-terminal fusion with tags.
Significance (*P* value) was evaluated by two-sided *t*-test, *, **, *** denote *P* value < 0.05, <0.01, <0.001, respectively, NS, not significant (*P* > 0.05).
c: Mass spectrum of zosteric acid.
All the data are expressed as the mean ± S.D. from three (*n* = 3) biologically independent replicates. Source data are provided as a Source Data file.

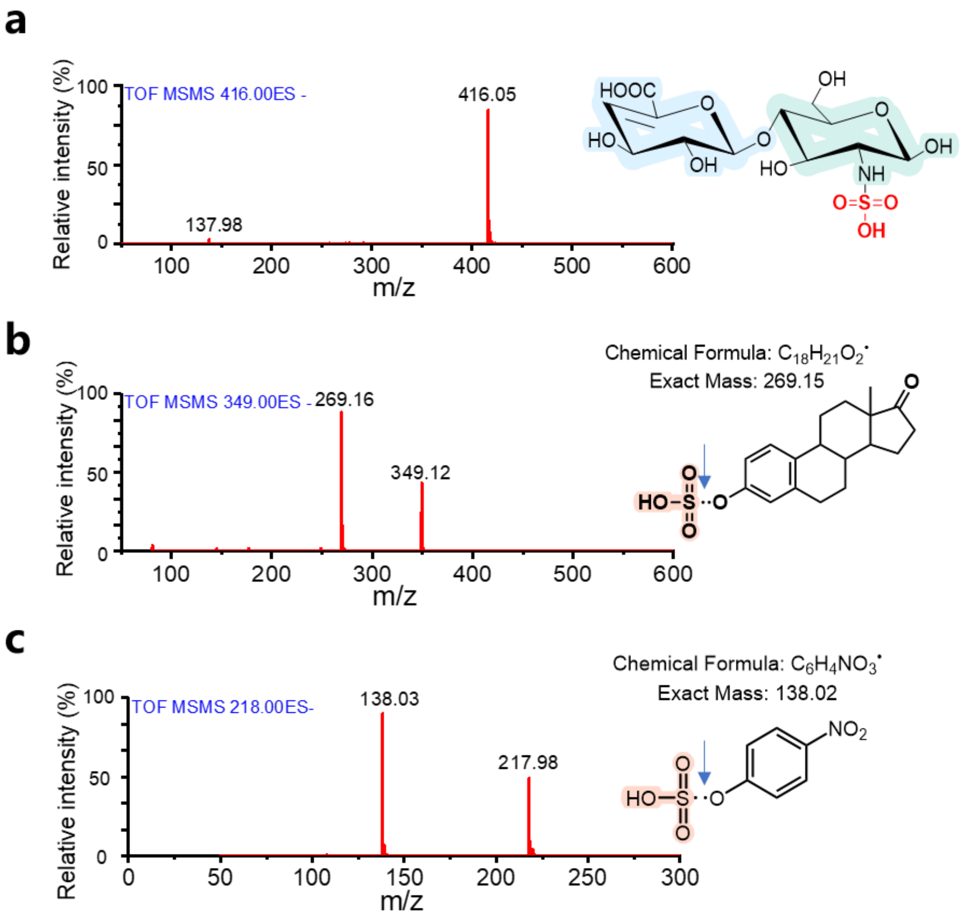

Supplementary Fig. 10. Mass spectrometry identification of different sulfonation products.

a: Mass spectrum identification of N-sulfated heparin disaccharide.
b: Mass spectrum identification of estrogen sulfate.
c: Mass spectrum identification of PNPS.

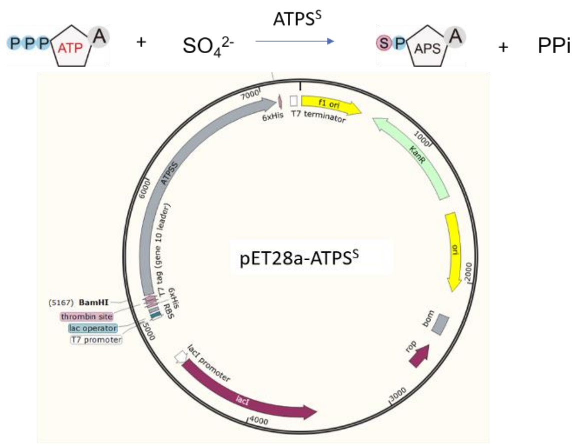

Supplementary Fig. 11. The plasmid construction schematic and sequences of pET28a-ATPSS for APS biosynthesis.

The sequences are provided as a supplementary Data 3 in Excel format.

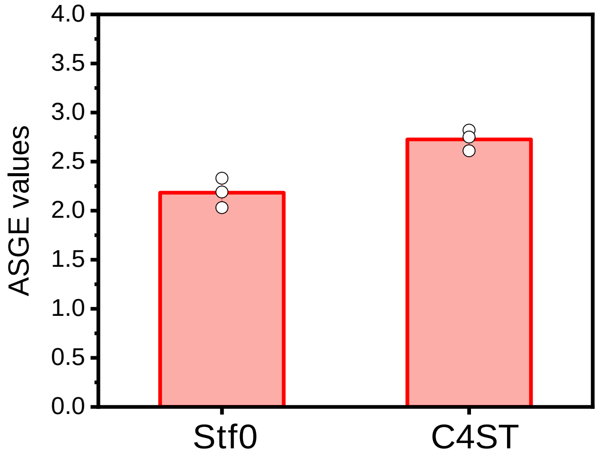

Supplementary Fig. 12. Quantification the active sulfate group equivalent values of Stf0 and C4ST coupling with the APS regeneration system.

All the data are expressed as the mean ± S.D. from three (n = 3) biologically independent replicates.

Source data are provided as a Source Data file.

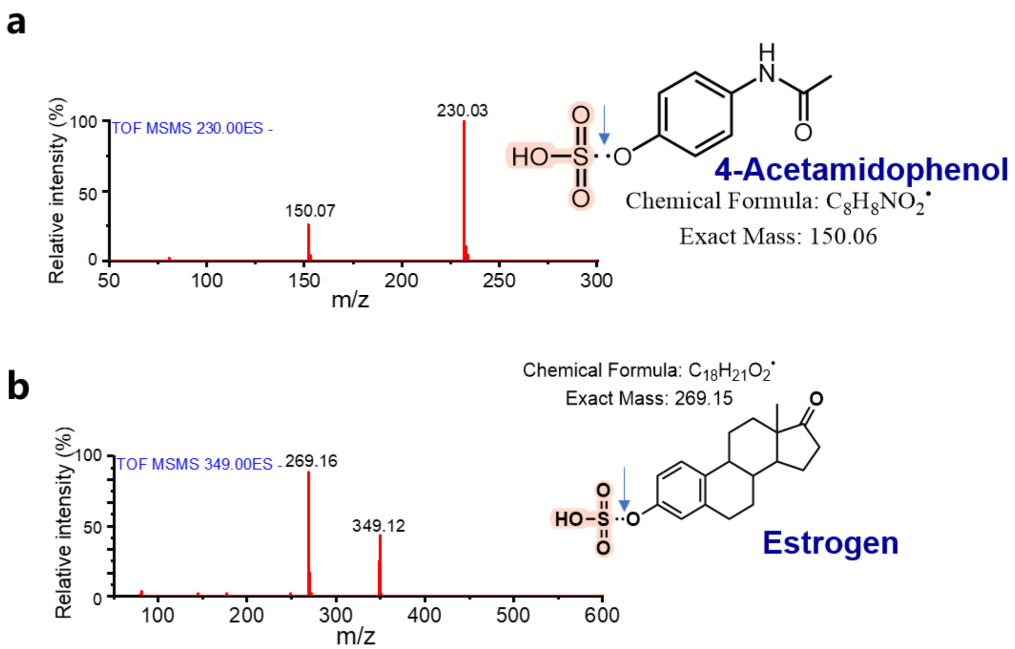

Supplementary Fig. 13. Mass spectrometry identification of different sulfonation products.

a: Mass spectrum identification of sulfated 4-acetamidophenol.

b: Mass spectrum identification of estrone sulfate.

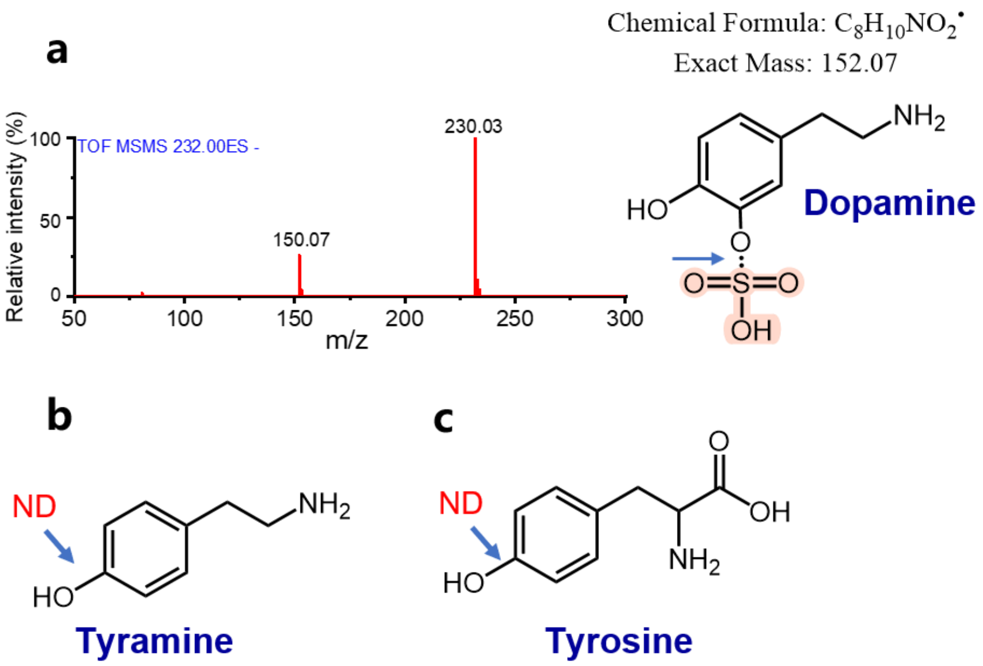

Supplementary Fig. 14. Mass spectrometry identification of different sulfonation products.

a: Mass spectrum identification of sulfated dopamine.
b: Structural formula of tyramine. ND means sulfation at the designated position was not detected.
c: Structural formula of tyrosine. ND means sulfation at the designated position was not detected.

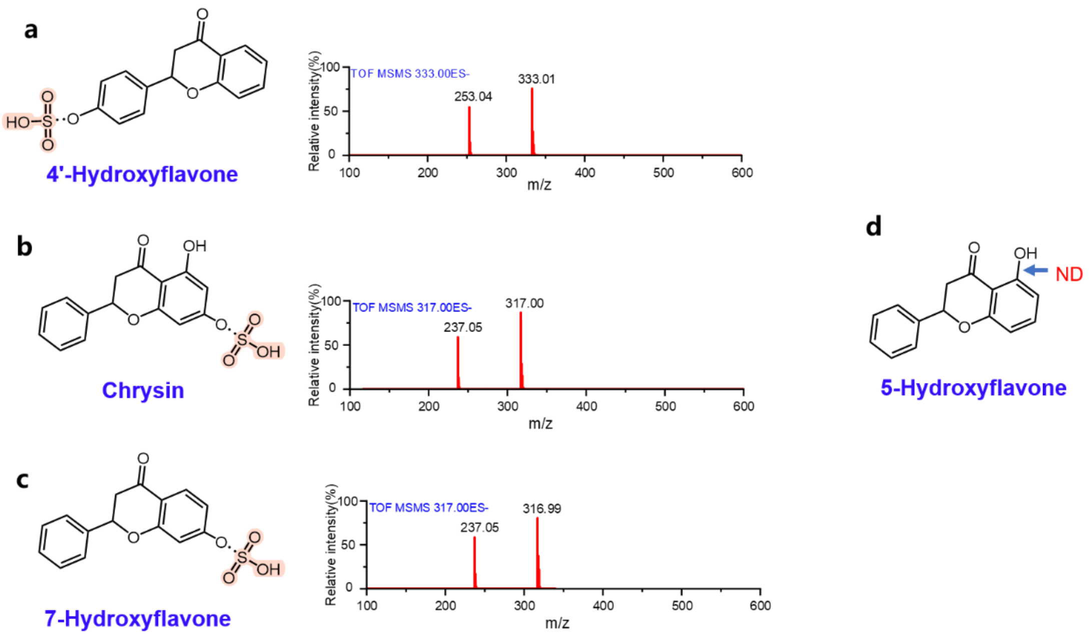

Supplementary Fig. 15. Mass spectrometry identification of sulfonation sites of different flavonoids.

a: Mass spectrum of sulfated 4'-hydroxyflavone.
b: Mass spectrum of sulfated chrysin.
c: Mass spectrum of sulfated 7-hydroxyflavone.
d: Structural formula of 5-hydroxyflavone. ND means sulfation at the designated position was not detected.

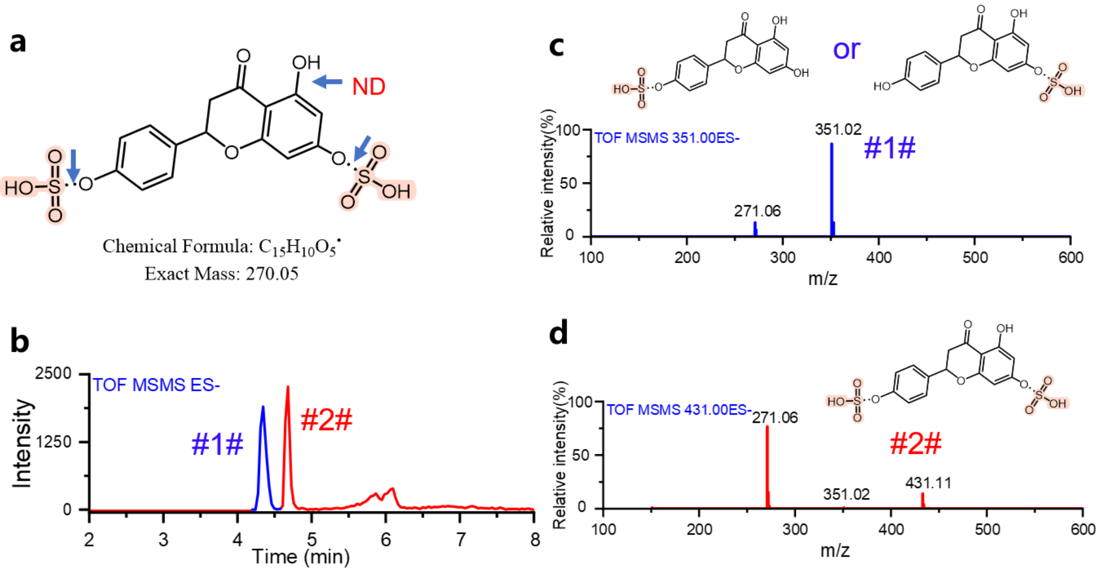

Supplementary Fig. 16. Identification of naringenin sulfonation pattern.

a: Structural formula of naringenin sulfates. ND means sulfation at the designated position was not detected.

Extracted ion chromatogram (b) and mass spectrum (c) of single site sulfated naringenin and (d) of two sites sulfated naringenin analyzed by HPLC-MS.

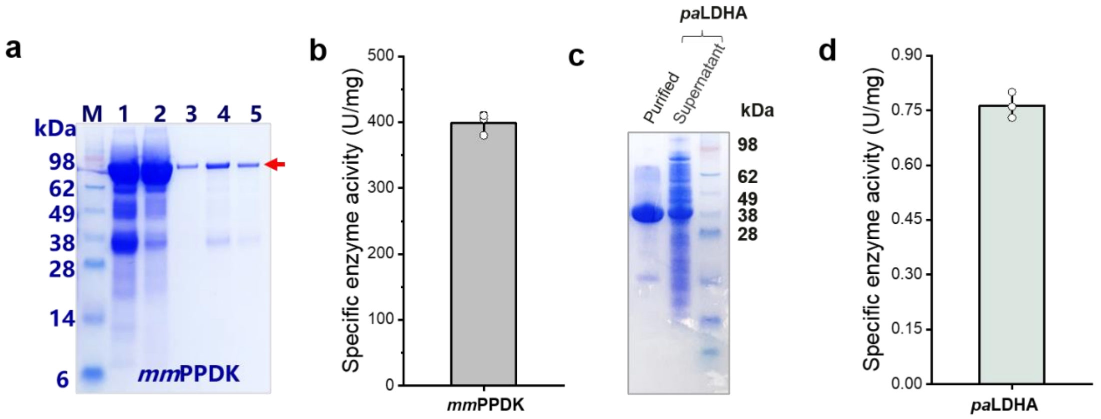

Supplementary Fig. 17. Expression and enzyme activity assay of *mm*PPDK and *pa*LDHA.

a: SDS-PAGE analysis of the expression and partial purification of *mm*PPDK. Different lanes represent gradient elution of purified *mm*PPDK, arrows indicate target bands. Three independent experiments were performed, with similar results.

b: Determination of specific enzyme activity of *mm*PPDK.

c: SDS-PAGE analysis of the expression and partial purification of lactate dehydrogenase *pa*LDHA from *Pseudomonas aeruginosa* PAO1.

Three independent experiments were performed, with similar results.

d: Determination of specific enzyme activity of *pa*LDHA.

All the data are expressed as the mean ± S.D. from three (n = 3) biologically independent replicates. Source data are provided as a Source Data file.

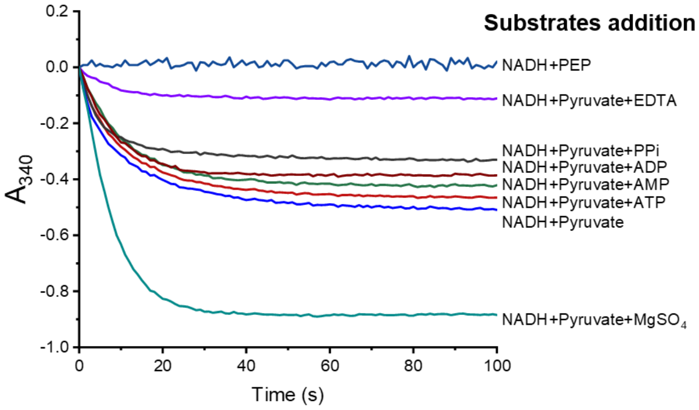

Supplementary Fig. 18. Analysis of the effect of substrates on *pa*LDAH in APS-sulfonation catalytic system. Source data are provided as a Source Data file.

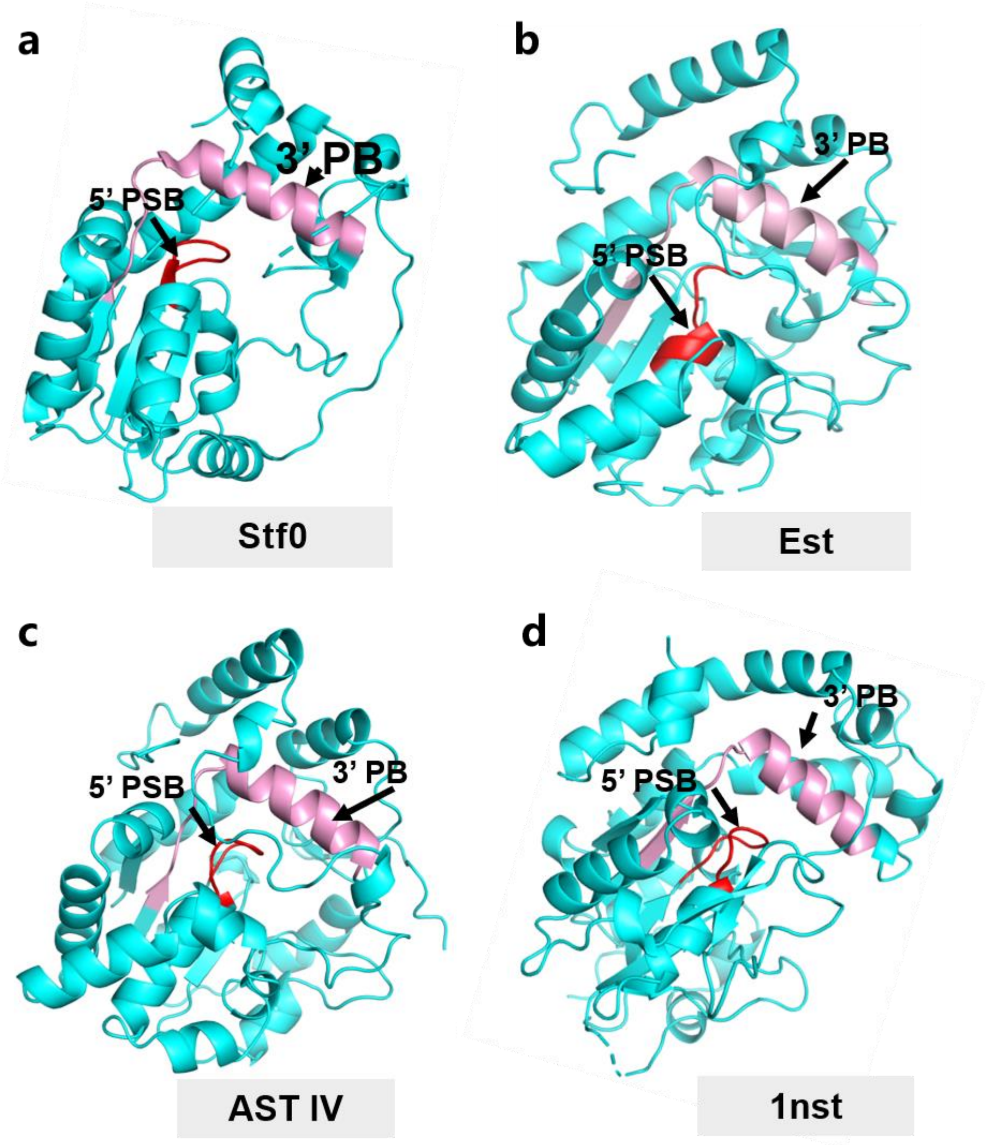

Supplementary Fig. 19. 3D Location of 3'PB and 5'PSB of sulfotransferases.

a: The structure of Stf0[5].
(https://www.rcsb.org/structure/1TEX)
b: The structure of EST, estrogen of sulfotransferases[6] .
(https://www.rcsb.org/structure/1BO6)
c: The structure of rat sulfotransferases IV[7].
(https://www.rcsb.org/structure/4P05)
d: The structure of human heparin N-sulfotransferase[8].
(https://www.rcsb.org/structure/1NST).

Supplementary references

1. Jin X*, et al.* Optimizing the sulfation-modification system for scale preparation of chondroitin sulfate A. *Carbohydr Polym* **246**, 116570 (2020).
2. Datta P, Fu L, He W, Koffas MAG, Dordick JS, Linhardt RJ. Expression of enzymes for 3'-phosphoadenosine-5'-phosphosulfate (PAPS) biosynthesis and their preparation for PAPS synthesis and regeneration. *Appl Microbiol Biotechnol* **104**, 7067-7078 (2020).
3. Burkart MD, Izumi M, Chapman E, Lin CH, Wong CH. Regeneration of PAPS for the enzymatic synthesis of sulfated oligosaccharides. *J Org Chem* **65**, 5565-5574 (2000).
4. Badri A, Williams A, Xia K, Linhardt RJ, Koffas MAG. Increased 3'-phosphoadenosine-5'-phosphosulfate levels in engineered *Escherichia coli* cell lysate facilitate the *in vitro* synthesis of chondroitin sulfate A. *Biotechnol J* **14**, e1800436 (2019).
5. Mougous JD*, et al.* Identification, function and structure of the mycobacterial sulfotransferase that initiates sulfolipid-1 biosynthesis. *Nat Struct Mol Biol* **11**, 721-729 (2004).
6. Kakuta Y, Pedersen LG, Carter CW, Negishi M, Pedersen LC. Crystal structure of estrogen sulphotransferase. *Nat Struct Biol* **4**, 904-908 (1997).
7. Zhou Z, Li Q, Xu R, Wang B, Du G, Kang Z. Secretory expression of the rat aryl sulfotransferases IV with improved catalytic efficiency by molecular engineering. *3 Biotech* **9**, 246 (2019).
8. Kakuta Y, Sueyoshi T, Negishi M, Pedersen LC. Crystal structure of the sulfotransferase domain of human heparan sulfate N-deacetylase/ N-sulfotransferase 1. *The Journal of Biological Chemistry* **274**, 10673-10676 (1999).
