# Supplementary material for: Engineering sulfonate group donor regeneration systems to boost biosynthesis of sulfated compounds
Source: Nat Commun. 2023 Nov 10;14:7297. doi: 10.1038/s41467-023-43195-1 (PMC10638397; doi:10.1038/s41467-023-43195-1)
Supplement: Supplementary file 3 — Description of Additional Supplementary Files [file 41467_2023_43195_MOESM3_ESM.pdf]

# Description of Additional Supplementary Files

**File Name:** Supplementary Data 1
**Description:** Strains and plasmids used in this study.

**File Name:** Supplementary Data 2
**Description:** Primers used in this study.

**File Name:** Supplementary Data 3
**Description:** The sequences of plasmid pET28a-ATPS$^{S}$.
