# Supplementary material for: Engineering sulfonate group donor regeneration systems to boost biosynthesis of sulfated compounds
Source: Nat Commun. 2023 Nov 10;14:7297. doi: 10.1038/s41467-023-43195-1 (PMC10638397; doi:10.1038/s41467-023-43195-1)
Supplement: Supplementary file 7 — Reporting Summary [file 41467_2023_43195_MOESM7_ESM.pdf]

nature portfolio

Corresponding author(s): Zhen Kang

Last updated by author(s): Oct 27, 2023

Reporting Summary

Nature Portfolio wishes to improve the reproducibility of the work that we publish. This form provides structure for consistency and transparency in reporting. For further information on Nature Portfolio policies, see our Editorial Policies and the Editorial Policy Checklist.

Statistics

For all statistical analyses, confirm that the following items are present in the figure legend, table legend, main text, or Methods section.

n/a	Confirmed	
☐	☒	The exact sample size (*n*) for each experimental group/condition, given as a discrete number and unit of measurement
☐	☒	A statement on whether measurements were taken from distinct samples or whether the same sample was measured repeatedly
☐	☒	The statistical test(s) used AND whether they are one- or two-sided<br>*Only common tests should be described solely by name; describe more complex techniques in the Methods section.*
☒	☐	A description of all covariates tested
☒	☐	A description of any assumptions or corrections, such as tests of normality and adjustment for multiple comparisons
☐	☒	A full description of the statistical parameters including central tendency (e.g. means) or other basic estimates (e.g. regression coefficient) AND variation (e.g. standard deviation) or associated estimates of uncertainty (e.g. confidence intervals)
☒	☐	For null hypothesis testing, the test statistic (e.g. *F*, *t*, *r*) with confidence intervals, effect sizes, degrees of freedom and *P* value noted<br>*Give P values as exact values whenever suitable.*
☒	☐	For Bayesian analysis, information on the choice of priors and Markov chain Monte Carlo settings
☒	☐	For hierarchical and complex designs, identification of the appropriate level for tests and full reporting of outcomes
☒	☐	Estimates of effect sizes (e.g. Cohen's *d*, Pearson's *r*), indicating how they were calculated

Our web collection on statistics for biologists contains articles on many of the points above.

Software and code

Policy information about availability of computer code

Data collection	MassLynx 4.1 and Empower 3 were used for collecting enzyme activity data. Discovery Studio 3 was for molecular docking. NCBI Nucleotide Database, NCBI Protein Database , NCBI Nucleotide Blast, NCBI Protein Blast and UniProt were used for collecting the gene and protein sequences, .
Data analysis	GraphPad Prism 8, Origin 2021, Adobe Illustrator 2020, Microsoft Office, ChemDraw 2020 and PyMOl were used for date analysis and image display.

For manuscripts utilizing custom algorithms or software that are central to the research but not yet described in published literature, software must be made available to editors and reviewers. We strongly encourage code deposition in a community repository (e.g. GitHub). See the Nature Portfolio guidelines for submitting code & software for further information.

Data

Policy information about availability of data

All manuscripts must include a data availability statement. This statement should provide the following information, where applicable:

- Accession codes, unique identifiers, or web links for publicly available datasets
- A description of any restrictions on data availability
- For clinical datasets or third party data, please ensure that the statement adheres to our policy

The authors declare that all data supporting the findings of this study are available within the paper and its supplementary information files, or the data are also

available from the corresponding author upon reasonable request. A reporting summary for this Article is available as a Supplementary Information file. The datasets generated and analyzed during the current study are available from the corresponding author upon request. The source data were provided as a Supplementary Data.

Research involving human participants, their data, or biological material

Policy information about studies with human participants or human data. See also policy information about sex, gender (identity/presentation), and sexual orientation and race, ethnicity and racism.

Reporting on sex and gender	N/A
Reporting on race, ethnicity, or other socially relevant groupings	N/A
Population characteristics	N/A
Recruitment	N/A
Ethics oversight	N/A

Note that full information on the approval of the study protocol must also be provided in the manuscript.

Field-specific reporting

Please select the one below that is the best fit for your research. If you are not sure, read the appropriate sections before making your selection.

☒ Life sciences ☐ Behavioural & social sciences ☐ Ecological, evolutionary & environmental sciences

For a reference copy of the document with all sections, see nature.com/documents/nr-reporting-summary-flat.pdf

Life sciences study design

All studies must disclose on these points even when the disclosure is negative.

Sample size	No specific calculations were used to determine sample size for each experiment. This experiment was performed on bacteria cultivation in shake flasks and then for enzyme purification. Sample size was 3 for all shake flasks cultivation, 3 for enzyme activity assay. All experiments were performed with sample sizes of n = 3 unless otherwise state.
Data exclusions	No data was excluded.
Replication	Each experiment was performed with at least three biologically independent replicates to ensure reliability. We have encountered no problems with reproducibility.
Randomization	Single colonies were picked randomly and propagated as bacterial cultures and for next enzyme purification and activity assay. Therefore, randomization was not relevant.
Blinding	The analysis was carried out entirely on bacteria. All samples, cultures, and replicates were treated identically. Therefore, randomization was not relevant.<br>This study reports objective measurements of samples derived from bacterial culture, purified proteins, or chemical reactions. In each experiment, the samples were treated identically. Therefore, blinding was not relevant.

Reporting for specific materials, systems and methods

We require information from authors about some types of materials, experimental systems and methods used in many studies. Here, indicate whether each material, system or method listed is relevant to your study. If you are not sure if a list item applies to your research, read the appropriate section before selecting a response.

Materials & experimental systems

n/a	Involved in the study
☒	☐ Antibodies
☒	☐ Eukaryotic cell lines
☒	☐ Palaeontology and archaeology
☒	☐ Animals and other organisms
☒	☐ Clinical data
☒	☐ Dual use research of concern
☒	☐ Plants

Methods

n/a	Involved in the study
☒	☐ ChIP-seq
☒	☐ Flow cytometry
☒	☐ MRI-based neuroimaging
